# Serotonergic–Muscarinic Interaction within the Prefrontal Cortex as a Novel Target to Reverse Schizophrenia-Related Cognitive Symptoms

**DOI:** 10.3390/ijms22168612

**Published:** 2021-08-10

**Authors:** Paulina Cieślik, Adrianna Radulska, Grzegorz Burnat, Leszek Kalinowski, Joanna M. Wierońska

**Affiliations:** 1 Department of Neurobiology, Maj Institute of Pharmacology, Polish Academy of Sciences, 12 Smętna Street, 31-343 Kraków, Poland; burnat@if-pan.krakow.pl; 2 Department of Medical Laboratory Diagnostics—Fahrenheit Biobank BBMRI.pl, Medical University of Gdańsk, 7 Dębinki Street, 80-211 Gdańsk, Poland; adrianna.radulska@gumed.edu.pl (A.R.); leszek.kalinowski@gumed.edu.pl (L.K.); 3 Biobanking and Biomolecular Resources Research Infrastructure Consortium Poland (BBMRI.pl), 7 Dębinki Street, 80-211 Gdańsk, Poland; 4 BioTechMed Centre, Department of Mechanics of Materials and Structures, University of Technology, 11/12 Narutowicza Street, 80-223 Gdańsk, Poland

**Keywords:** schizophrenia, cognitive deficits, muscarinic receptors, M_1_, M_4_, M_5_, 5-HT_1A_

## Abstract

Recent studies revealed that the activation of serotonergic 5-HT_1A_ and muscarinic M_1_, M_4_, or M_5_ receptors prevent MK-801-induced cognitive impairments in animal models. In the present study, the effectiveness of the simultaneous activation of 5-HT_1A_ and muscarinic receptors at preventing MK-801-induced cognitive deficits in novel object recognition (NOR) or Y-maze tests was investigated. Activators of 5-HT_1A_ (F15599), M_1_ (VU0357017), M_4_ (VU0152100), or M_5_ (VU0238429) receptors administered at top doses for seven days reversed MK-801-induced deficits in the NOR test, similar to the simultaneous administration of subeffective doses of F15599 (0.05 mg/kg) with VU0357017 (0.15 mg/kg), VU0152100 (0.05 mg/kg), or VU0238429 (1 mg/kg). The compounds did not prevent the MK-801-induced impairment when administered acutely. Their activity was less evident in the Y-maze. Pharmacokinetic studies revealed high brain penetration of F15599 (brain/plasma ratio 620%), which was detected in the frontal cortex (FC) up to 2 h after administration. Decreases in the brain penetration properties of the compounds were observed after acute administration of the combinations, which might have influenced behavioral responses. This negative effect on brain penetration was not observed when the compounds were administered repeatedly. Based on our results, prolonged administration of a 5-HT_1A_ activator with muscarinic receptor ligands may be effective at reversing cognitive decline related to schizophrenia, and the FC may play a critical role in this interaction.

## 1. Introduction

Cognitive disturbances accompany the majority of mental and neurodegenerative disorders, including schizophrenia. Deficits may appear premorbidly in children and adolescents or occur suddenly; they persist chronically and may worsen over time, affecting daily functioning [1,2,3]. Multiple cognitive domains are affected in patients with schizophrenia [1,4], and up to date remain treatment resistant, although cognitive deficits in some individuals may be ameliorated with atypical antipsychotics such as clozapine, olanzapine, or lurasidone [5]. In preclinical experiments with the use of rodent models of learning and memory (e.g., novel object recognition test—NOR) the administration of atypical neuroleptics reversed cognitive decline induced by the administration of open channel blockers (OCB) of NMDA receptor, such as MK-801 (also known as dizocilpine) or phencyclidine (PCP) [6,7,8,9]. Putatively, activation of the 5-HT_1A_ receptor by atypical neuroleptics is responsible for their efficacy in reversing cognitive symptoms associated with schizophrenia [10,11].

Based on these observations, the modulation of 5-HT_1A_ receptors alone or as an add-on therapy might be beneficial in treating cognitive deficits in patients with schizophrenia [12]. As shown in our earlier studies, the activation of 5-HT_1A_ receptors plays an essential role in the procognitive activity of activators of group II or III metabotropic glutamatergic receptors, such as LY487379 or LSP4-2022. Their activity was inhibited by the administration of WAY100635 (5-HT_1A_ antagonist) or enhanced by the administration of (R,S)-8-OH-DPAT (5-HT_1A_ agonist) [7,13,14,15].

Both WAY100635 and (R,S)-8-OH-DPAT act in a two-phase manner. Low doses of the compounds are presumed to act at the presynaptic 5-HT_1A_ receptors localized in the raphe, while higher doses act at both the presynaptic and postsynaptic sites. Activation of postsynaptic receptors by high doses of agonists (R,S)-8-OH-DPAT may result in the development of serotonergic syndrome (lower lip retraction, forepaw treading, and flat body posture) [16,17] and constitute the limitation of their use, especially since they disturb memory processes [18]. Therefore, the use of selective biased agonists might be a better option. The recently developed compound F15599 (also known as NLX-101) preferentially stimulates postsynaptic 5-HT_1A_ receptors in brain structures related to learning and memory processes, such as the frontal cortex and septo-hippocampal neurons, instead of dorsal raphe neurons [19,20].

In our previous studies, selected muscarinic ligands (VU0357017—M_1_ allosteric agonist, VU0152100—M_4_ positive allosteric modulator (PAM), and VU0238429—M_5_ PAM) ameliorated MK-801-induced deficits in tests assessing cognition, such as the NOR test or prepulse inhibition [21,22,23]. The action of the compounds was shown to be enhanced with the administration of metabotropic glutamatergic (mGlu) or GABA_B_ receptor activators [21,22,23,24].

**Figure 1:**
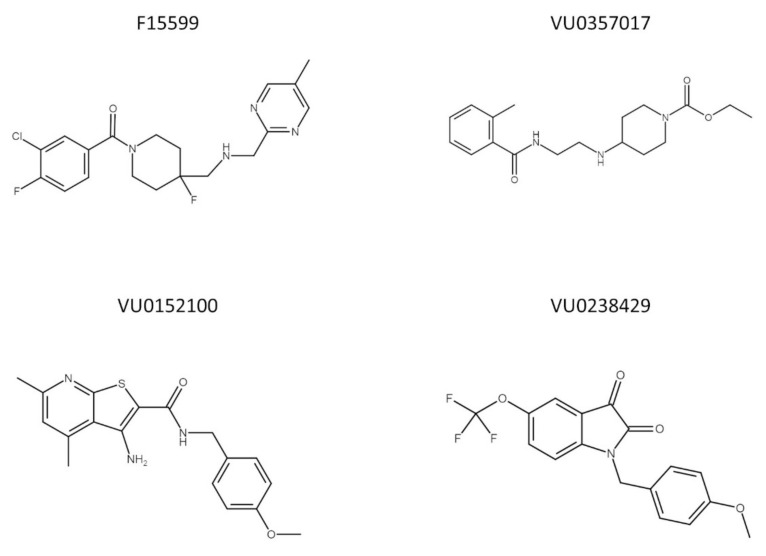
Chemical structure of F15599, VU0357017, VU0152100, and VU0238429.

To date, no data are available concerning the interaction between muscarinic and 5-HT_1A_ receptors, which is potentially interesting because of their overlapping expression in the structures involved in learning and memory processes. In the present study, the effectiveness of the simultaneous administration of muscarinic ligands (VU0357017, VU0152100, and VU0238429) with a biased agonist of 5-HT_1A_ receptors (F15599) (the chemical structures of the compounds are presented on Figure 1) at reversing schizophrenia-related cognitive disturbances was examined in behavioral tests assessing memory processes (NOR test and Y-maze). The potential of the combinations to prevent or reverse MK-801-induced deficits was examined after acute or chronic administration of the tested compounds. Pharmacokinetic studies were performed to exclude any potential drug–drug interactions influencing the brain penetration properties of the compounds. The analysis was performed in the frontal cortex both after the administration of the drugs alone at the top doses and combinations at subeffective doses based on behavioral studies. The compounds were administered either acutely or repeatedly.

## 2. Results

### 2.1. Treatment Regimen

**Figure 2:**
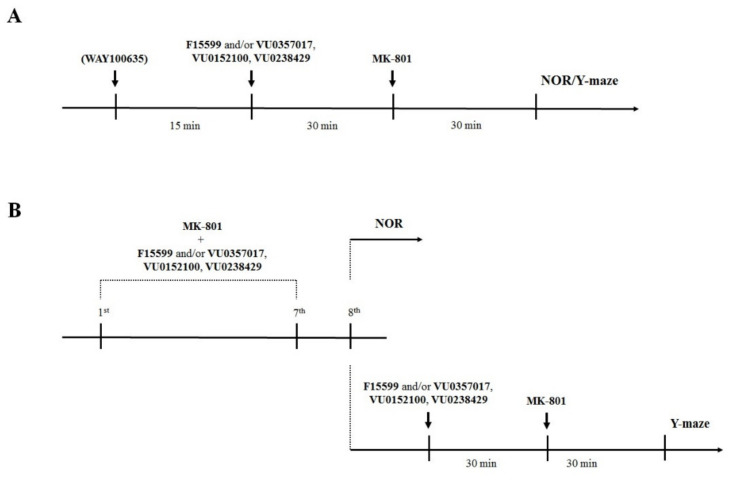
Schematic representation of the experiments performed in this study. (**A**) Acute administration of the investigated compounds and MK-801. (**B**) Prolonged administration of the investigated compounds and MK-801.

Drugs were administered on two different schedules: (1) acute administration of compounds 45 min (WAY100635—5-HT_1A_ antagonist) or 30 min (VU0357017, VU152100, VU0238429, and F15599) before MK-801 (also administered once 30 min before the test in Y-maze or T_1_ in NOR test); and (2) prolonged administration (once per day for 7 consecutive days) of the compounds and MK-801 with the last administration 24 h before the test (NOR test) or at the day of the test (Y-maze). This difference was due to the lack of MK-801 disruptive effect on animals’ behavior in Y-maze when the test was performed 24 h after the last administration. Schedules of administration are presented graphically in Figure 2. The subeffective doses of the compounds for acute administration in the NOR test were chosen based on our previous research (see: [22,23]) while dose-dependent studies of the activity of the compounds in reversing disruptions in the Y-maze or after prolonged administration along with MK-801 were performed within this study. Different vehicles were used throughout the study: (1) 0.9% NaCl and (2) 10% Tween 80. Vehicles were administered to all animals (e.g., control mice and MK-801-treated mice) on the same schedule as drugs were administered to the appropriate drug-treated groups of mice. Neither solvent exerted effects on animal behavior.

### 2.2. Acute Administration

#### 2.2.1. Activity of F15599 in NOR and Y-Maze Test

MK-801 induced typical disruption in the recognition index (at least *p* < 0.01) and spontaneous alternations in the Y-maze (*p* < 0.05).

**Figure 3:**
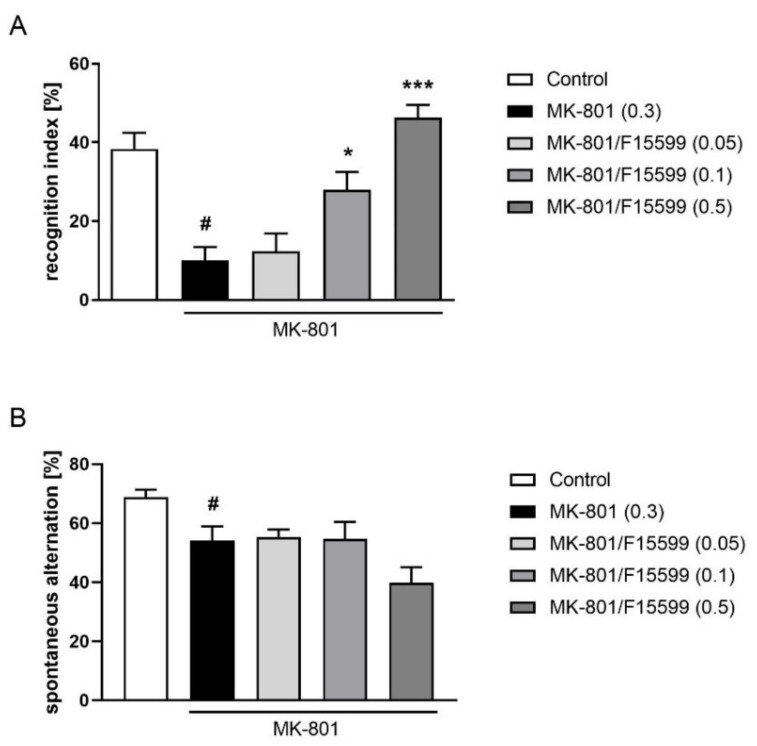
Activity of F15599 in the NOR (**A**) or Y-maze (**B**) test. The data are presented as means ± SEM. Doses of the compounds are indicated in parentheses. # *p* < 0.001 (**A**) or # *p* < 0.01 (**B**) compared to control animals and * *p* < 0.05, *** *p* < 0.001 compared to MK-801-treated mice.

The administration of F15599 at 0.1 or 0.5 mg/kg reversed the MK-801-induced deficits in the NOR test (F_(3,30)_ = 14.79, *p* < 0.001) (Figure 3A). The dose of 0.05 mg/kg was ineffective. F15599 administered at doses of 0.05–0.5 mg/kg showed no efficacy in the Y-maze test (Figure 3B).

#### 2.2.2. Activity of Muscarinic Activators in the Y-Maze Test

**Figure 4:**
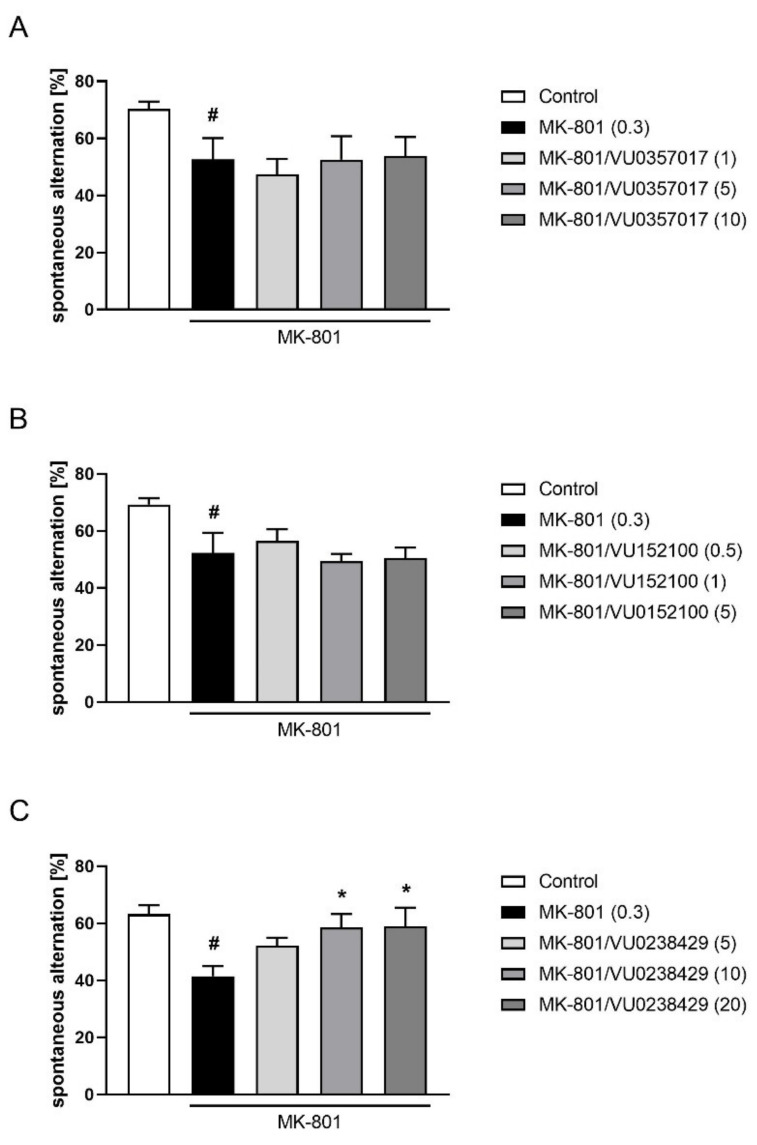
Activity of VU0357017 (**A**), VU0152100 (**B**), and VU0238429 (**C**) in the Y-maze test. The data are presented as means ± SEM. Doses of the compounds are indicated in parentheses. # *p* < 0.05 (**A**,**B**) or # *p* < 0.001 (**C**) compared to control animals and * *p* < 0.05 compared to MK-801-treated mice.

No efficacy was observed for VU0357017 administered at doses of 1–10 mg/kg or VU0152100 administered at doses of 0.1–5 mg/kg. VU0238429 reversed the reduction in spontaneous alternations in the Y-maze induced by MK-801 (F_(3,34)_ = 3.372, *p* < 0.05) at doses of 10 and 20 mg/kg. The dose of 5 mg/kg was ineffective (Figure 4).

#### 2.2.3. Combined Administration of Muscarinic Activators and WAY100635 in NOR Test

**Figure 5:**
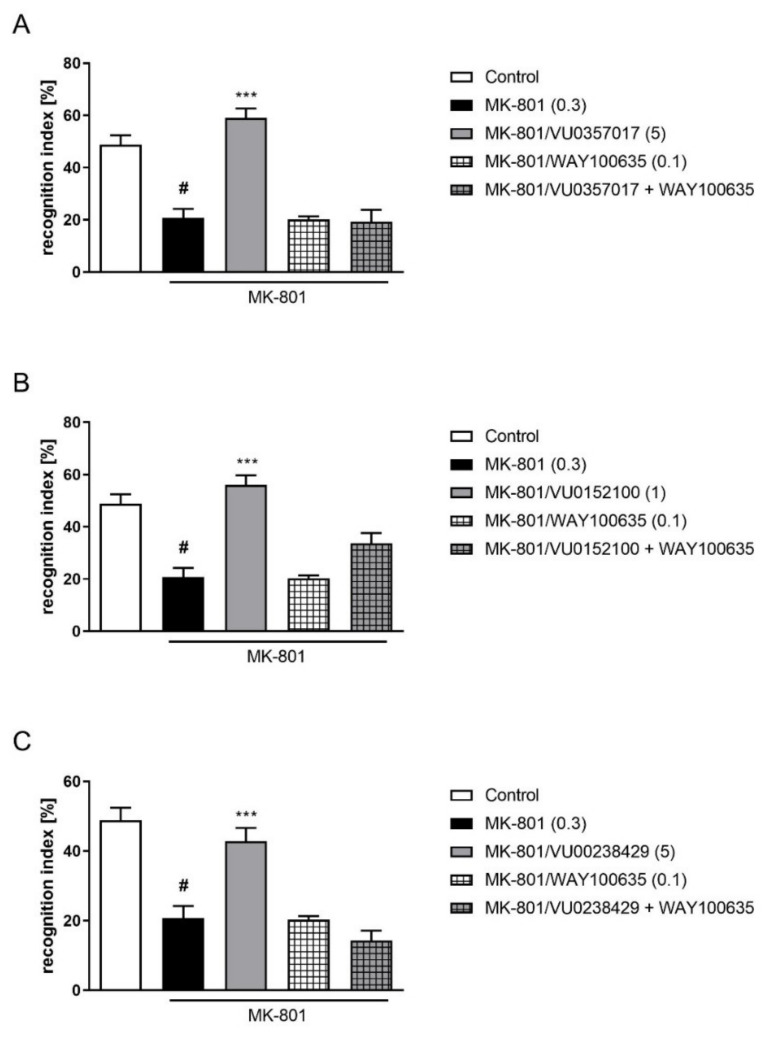
Activity of the combined administration of WAY100635 with active dose of VU0357017 (**A**), VU0152100 (**B**), or VU0238429 (**C**) in the NOR test. The data are presented as means ± SEM. Doses of the compounds are indicated in parentheses. # *p* < 0.001 compared to control animals and *** *p* < 0.001 compared to MK-801-treated mice.

The administration of WAY100635 at a dose of 0.1 mg/kg 15 min before VU0357017 (5 mg/kg), VU0152100 (1 mg/kg), or VU0238429 (5 mg/kg) reversed the pro-cognitive effect of the administration of the top doses of the ligands (F_(1,29)_ = 33.8087, *p* < 0.001; F_(1,33)_ = 10.7161, *p* < 0.01; F_(1,25)_ = 23.7360, *p* < 0.001, respectively) (Figure 5).

#### 2.2.4. Combined Administration of Muscarinic Activators and F15599 at Subeffective Doses in NOR and Y-Maze Test

**Figure 6:**
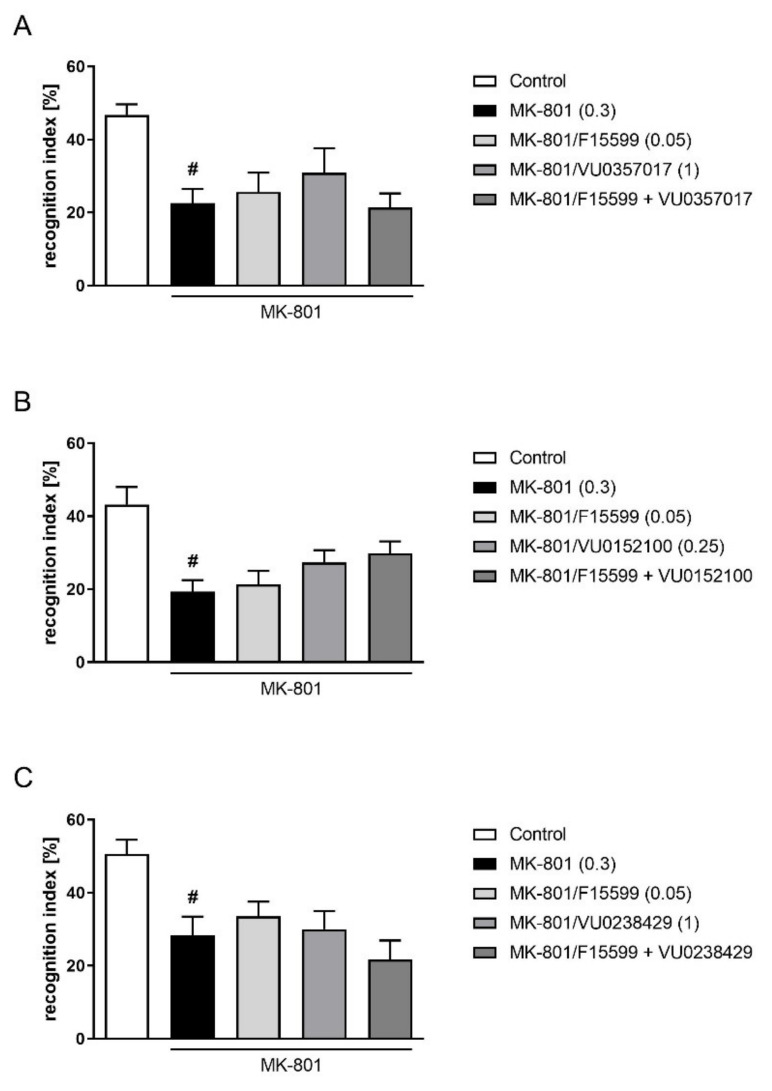
Activity of the combined administration of subeffective dose of F15599 with subeffective dose of VU0357017 (**A**), VU0152100 (**B**), or VU0238429 (**C**) in the NOR test. The data are presented as means ± SEM. Doses of the compounds are indicated in parentheses. # *p* < 0.001 (**A**,**B**) or # *p* < 0.01 (**C**) compared to control animals.

Combined administration of subeffective doses of F15599 with VU0357017, VU0152100, or VU0238429 had no effect on memory deficits induced by MK-801 administration in the NOR test (Figure 6).

**Figure 7:**
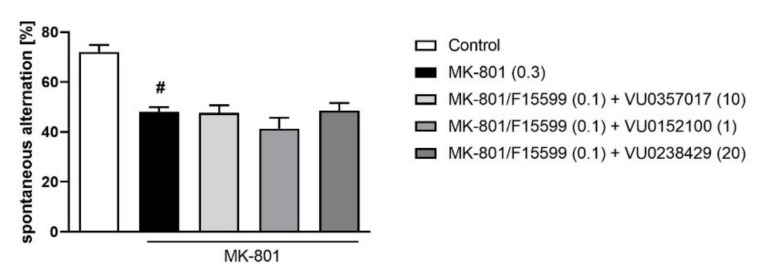
Activity of the combined administration of subeffective dose of F15599 with subeffective dose of VU0357017, VU0152100, or VU0238429 in the Y-maze test. The data are presented as means ± SEM. Doses of the compounds are indicated in parentheses. # *p* < 0.001 compared to control animals.

Combined administration of F15599 at a dose of 0.1 mg/kg together with VU0357017 (10 mg/kg), VU0152100 (1 mg/kg), or VU0238429 (5 mg/kg) had no effect on spontaneous alternations in the Y-maze test (Figure 7).

### 2.3. Chronic Administration

#### 2.3.1. Activity of F15599

Seven days of MK-801 administration induced a typical disruption in the recognition index (at least *p* < 0.01).

**Figure 8:**
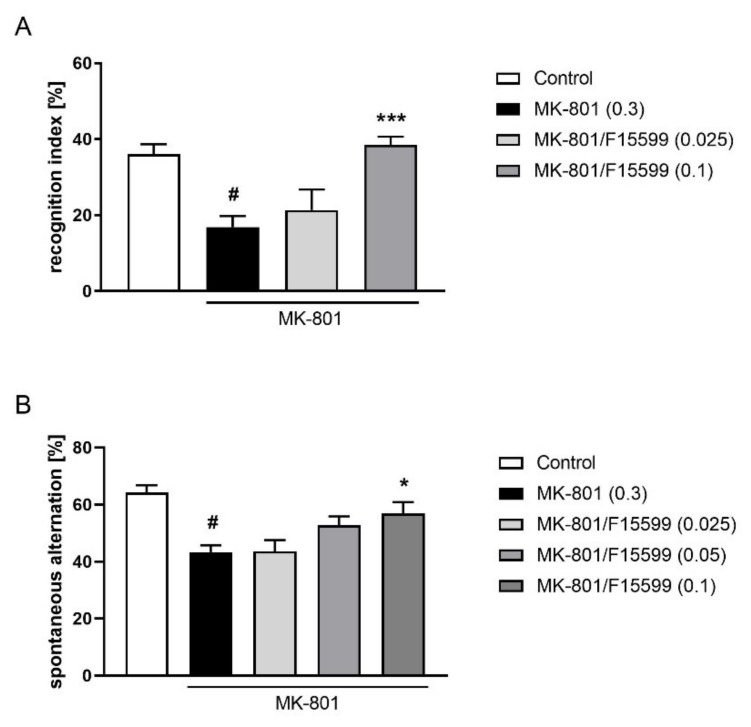
Activity of F15599 in the NOR (**A**) or Y-maze (**B**) test after seven days of treatment. The data are presented as means ± SEM. Doses of the compounds are indicated in parentheses. # *p* < 0.001 compared to control animals and * *p* < 0.05, *** *p* < 0.001 compared to MK-801-treated mice.

The administration of F15599 at a dose of 0.1 mg/kg reversed the reduction in the recognition index induced by MK-801 (F_(2,34)_ = 11.21, *p* < 0.001). The dose of 0.025 mg/kg was ineffective (Figure 8A).

At the highest dose tested (0.1 mg/kg), F15599 reversed the reduction in spontaneous alternations induced by MK-801 (F_(3,36)_ = 4.187; *p* < 0.05) (Figure 8B). The doses of 0.025 and 0.05 mg/kg were ineffective.

#### 2.3.2. Dose-Dependent Activity of Muscarinic Activators

**Figure 9:**
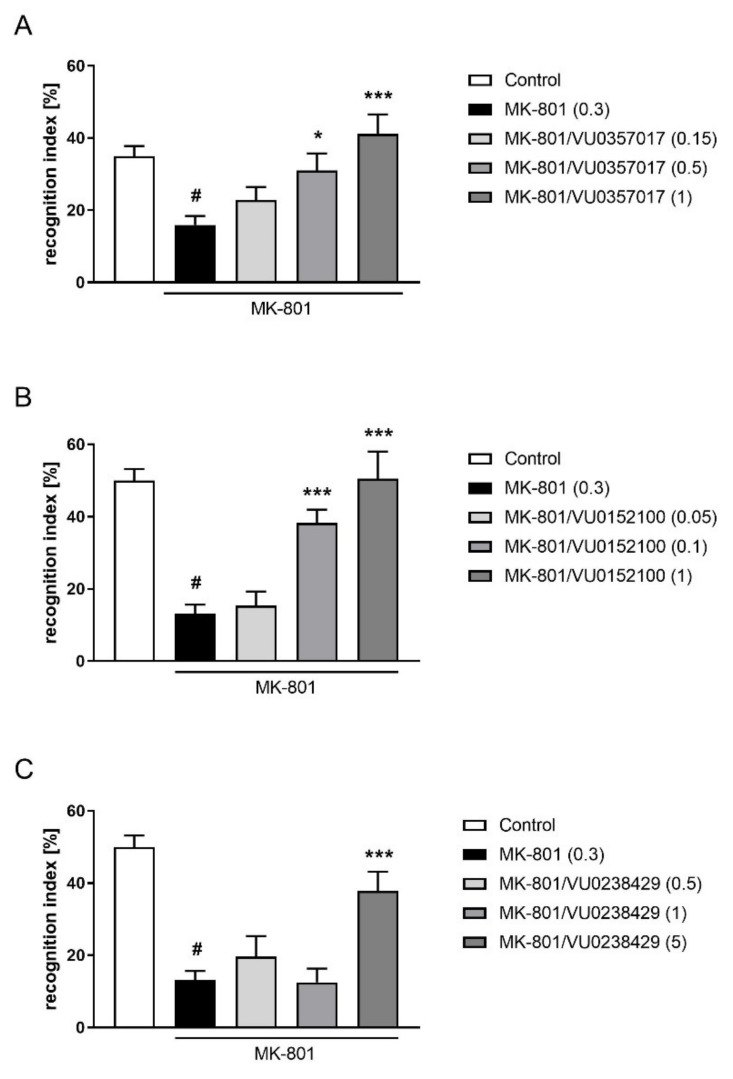
Activity of VU0357017 (**A**), VU0152100 (**B**), and VU0238429 (**C**) in the NOR test after seven days of administration. The data are presented as means ± SEM. Doses of the compounds are indicated in parentheses. # *p* < 0.001 compared to control animals and * *p* < 0.05, *** *p* < 0.001 compared to MK-801-treated mice.

VU0357017 administered at doses ranging from 0.15 to 5 mg/kg ameliorated MK-801-induced deficits in the NOR test (F_(3,35)_ = 6.829; *p* < 0.001). The dose of 0.15 mg/kg was ineffective. The activity of VU0152100 was observed at doses of 0.1 and 1 mg/kg (F_(3,35)_ = 17.38; *p* < 0.001), and a dose of 0.05 was ineffective. VU0238429 was only active at a dose of 5 mg/kg (F_(3,35)_ = 8,067; *p* < 0.001), and doses of 0.5 and 1 mg/kg were ineffective (Figure 9).

**Figure 10:**
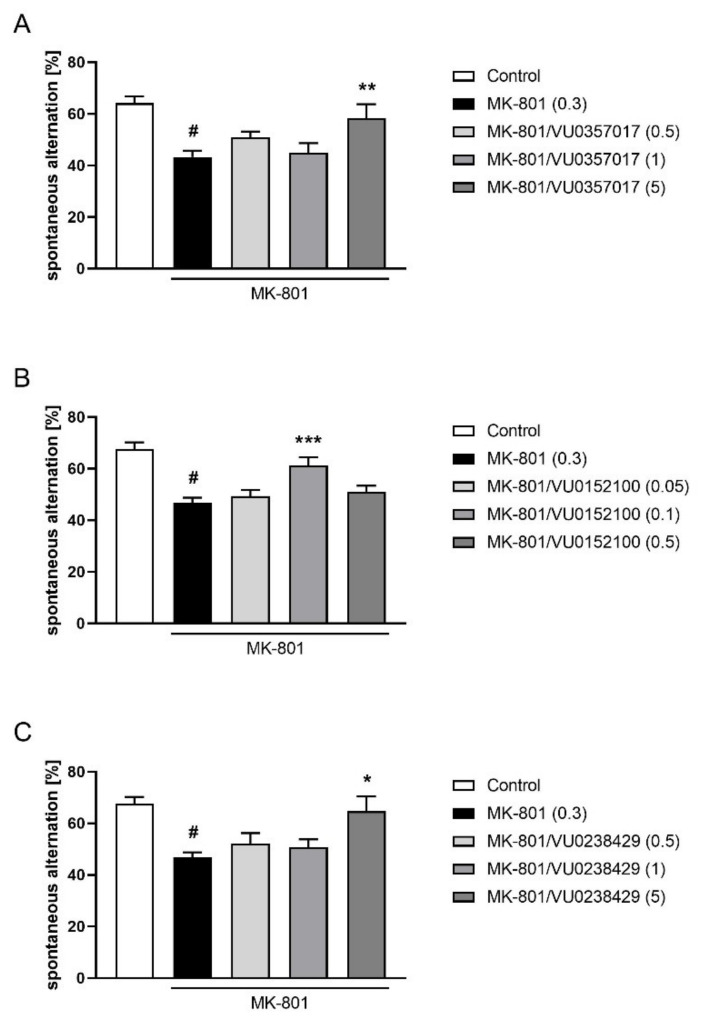
Activity of VU0357017 (**A**), VU0152100 (**B**), and VU0238429 (**C**) in the Y-maze test after seven days of administration. The data are presented as means ± SEM. Doses of the compounds are indicated in parentheses. # *p* < 0.001 compared to control animals and * *p* < 0.05, ** *p* < 0.01, *** *p* < 0.001 compared to MK-801-treated mice.

In the Y-maze test, the activity of VU0357017 was observed at a dose of 5 mg/kg (F_(3,36)_ = 3.795; *p* < 0.05) but not at 0.5 or 1 mg/kg. VU0152100 was only active at the dose 0.1 (F_(4,46)_ = 4.990; *p* < 0.01) and VU0238429 was active at a dose of 5 mg/kg (F_(3,37)_ = 4.579; *p* < 0.01) but not at 0.5 or 1 mg/kg (Figure 10).

#### 2.3.3. Combined Administration of Muscarinic Activators and F15599 at Subeffective Doses

**Figure 11:**
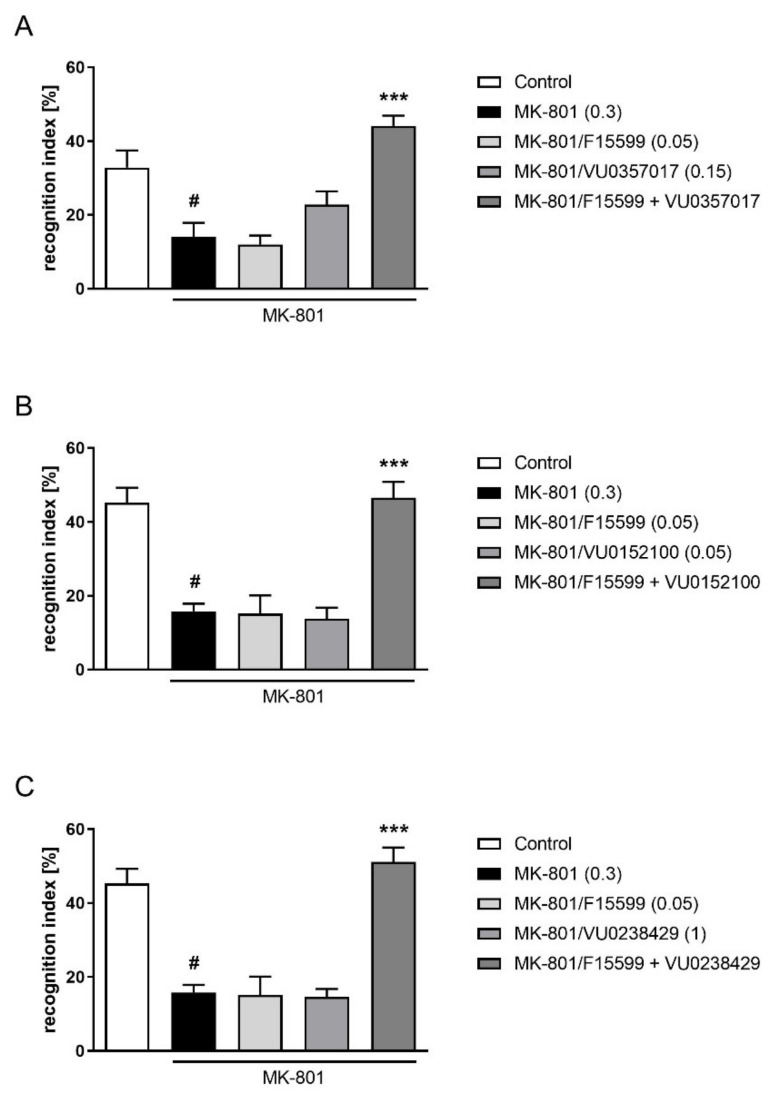
Activity of the combined administration of subeffective dose of F15599 with subeffective dose of VU0357017 (**A**), VU0152100 (**B**), or VU0238429 (**C**) in the NOR test after seven days of treatment. The data are presented as means ± SEM. Doses of the compounds are indicated in parentheses. # *p* < 0.01 (**A**) or # *p* < 0.001 (**B**,**C**) compared to control animals and *** *p* < 0.001 compared to MK-801-treated mice.

The combined administration of a subeffective dose of F15599 (0.05 mg/kg) with subeffective doses of VU0357017 (0.15 mg/kg), VU0152100 (0.05 mg/kg), or VU0238429 (1 mg/kg) reversed MK-801-induced disruptions in NOR test (F_(1,28)_ = 10.7384, *p* < 0.01; F_(1,31)_ = 17.2759, *p* < 0.001; F_(1,32)_ = 23.0644, *p* < 0.001, respectively) (Figure 11).

**Figure 12:**
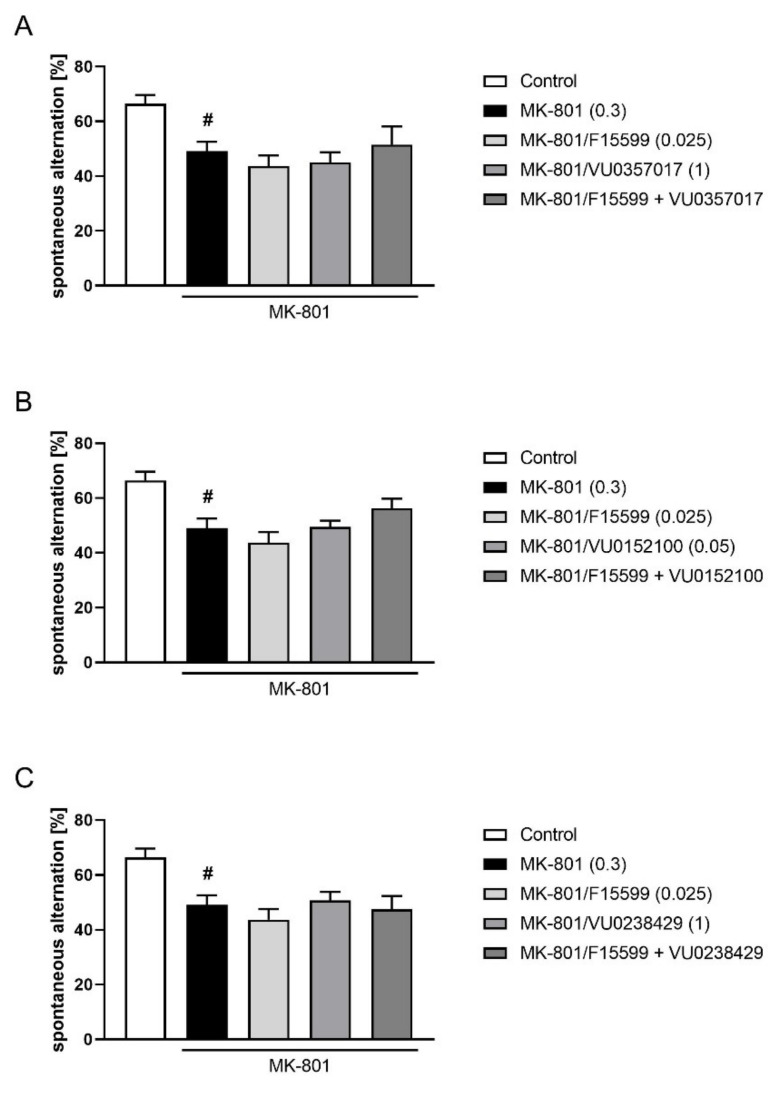
Activity of combined administration of subeffective dose of F15599 with subeffective dose of VU0357017 (**A**), VU0152100 (**B**), or VU0238429 (**C**) in the Y-maze test after seven days of treatment. The data are presented as means ± SEM. Doses of the compounds are indicated in parentheses. # *p* < 0.01 compared to control animals.

The combined administration of a subeffective dose of F15599 (0.025 mg/kg) with subeffective doses of VU0357017 (1 mg/kg), VU0152100 (0.05 mg/kg), or VU0238429 (1 mg/kg) did not reverse MK-801-induced disruptions in Y-maze (Figure 12).

### 2.4. Pharmacokinetic Studies

#### 2.4.1. Pharmacokinetic Analysis of F15599

**Figure 13:**
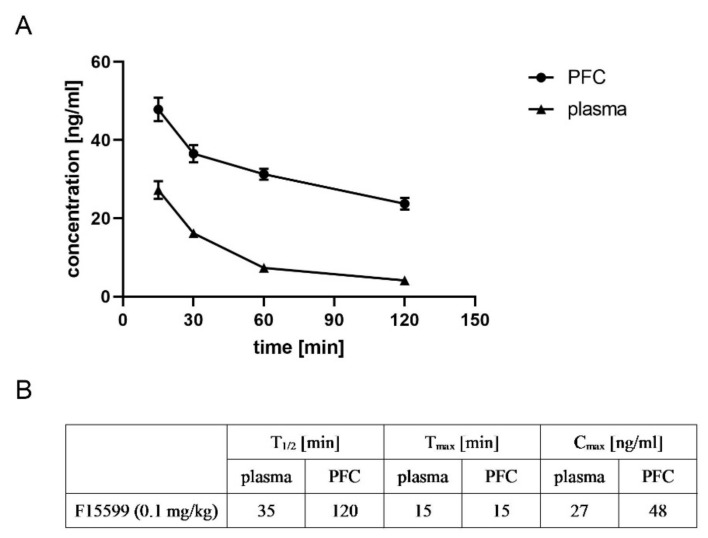
Pharmacokinetic analysis of the ability of F15599 to penetrate the frontal cortex. Time-dependent changes (**A**) and T_1/2_, T_max_ and C_max_ (**B**) are presented. F15599 was administered at a dose of 0.1 mg/kg.

F15599 reached its maximal concentration in both plasma and FC after 15 min (27 and 48 ng/mL, respectively). The drug was detected in plasma and brain up to 120 min after administration, and its concentration was still high (24 ng/mL in the brain). Time-dependent changes in the concentrations of F15599 and T_1/2_, T_max_, and C_max_ are presented in Figure 13.

#### 2.4.2. Pharmacokinetic Analysis of Drug–Drug Interactions

F15599 had the highest FC penetration among the tested ligands after acute administration at the top dose, reaching 620%. Prolonged administration of the compound decreased FC penetration by ca. 50%.

The percentage of FC penetration after acute administration of the compounds at the top doses alone was similar for VU0357017 and VU0238429 (67% and 53%, respectively). Only 7% of VU0152100 accumulated in the FC. Prolonged administration of the compounds to animals treated with MK-801 decreased the FC penetration ratio by ca. 50% for VU0357017 and VU0238429 (which was 36% and 21%, respectively), except for VU0152100 the penetration of which was almost 50% higher than after acute administration.

Acute, concomitant administration of subeffective doses of F15599 and VU0357017 or VU0238429 resulted in a decrease of FC penetration of VU0357017 (more than 50%), VU0238429 (~40%). Penetration of F15599 decreased ~50%, when administered simultaneously with VU0152100 or VU0238429. On the contrary, FC penetration of VU0152100 increased more than 50% when administered concomitantly with F15599.

Chronic administration of subeffective doses of the compounds increased the brain penetration of F15599, but had no effect on muscarinic ligands.

The drug concentrations in plasma and FC and the FC penetration level after acute or chronic administration of the tested compounds either at the highest doses alone or at subeffective doses in combination are presented in Table 1.

## 3. Discussion

The present study aimed to investigate the benefits of simultaneous activation of 5-HT_1A_ and muscarinic receptors after the combined administration of subeffective doses of their activators. To date, this study is the first to document the activity of a 5-HT_1A_-biased agonist, F15599, in tests assessing cognition and the first pharmacokinetic analysis showing the brain penetrating abilities of the compound.

The efficacy of F15599 in ameliorating MK-801-induced disruptions in NOR was evident both after acute and repeated administration, while in the Y-maze, the compound was active only after acute and not repeated administration. Previously it was shown that the compound ameliorated PCP-induced deficits during the reversal phase of the learning task and in the hole-board test [25]. In our subsequent studies in which the effectiveness of the simultaneous administration of F15599 with the activators of muscarinic receptors was investigated, doses of F15599 lower than 0.1 mg/kg were used, as at doses greater than 0.16 mg/kg, F15599 induced some of the adverse effects on rats related to serotonergic syndrome [19].

The experiments were a continuation of our previous research in which the synergistic actions of muscarinic and metabotropic glutamate receptor activation were observed in animal models of schizophrenia [21,22,23,24].

No synergistic activity of the combined, single administration of low, subeffective doses of F15599, together with subeffective doses of any muscarinic activator was noticed. The results are in contrast with the majority of our earlier studies in which the efficacy of combined administration of subeffective doses of the 5-HT_1A_ agonist (R,S)-8-OH-DPAT potentiated the procognitive activity of subeffective doses of several mGlu activators e.g., LSP4-2022, LY379268 [7,13,14,15]. In parallel experiments, the activity of muscarinic receptor activators was blocked by WAY100635, a 5-HT_1A_ receptor antagonist, indicating the 5-HT_1A_-dependent activity of VU0357017, VU0152100, and VU0238429.

Subsequently, the efficacy of F15599 and muscarinic receptors activators was observed after prolonged (7 days) administration of the compounds along with MK-801. Under this treatment regimen, the activity of drugs in NOR was evident at lower doses than after acute administration [21,22,23,24] and was also noticed in the Y-maze test indicating, that lower doses of the compounds are sufficient to prevent memory impairments induced by prolonged MK-801 administration. The simultaneous prolonged administration of subeffective doses of F15599 with VU03567017, VU0152100, and VU0238429 reversed MK-801-induced disruptions in NOR, but not in the Y-maze test.

Overall, our results indicate that the animals’ responses both to the amnestic potency of MK-801 and to the preventive effectivity of the tested compounds were evidently weaker in the Y-maze test than in the NOR test. To some extent, this result may be due to the activation of slightly different pathways in the brain that regulate animals’ performance on each test and different types of cognitive processes activated by these two tasks (spatial working memory in the Y-maze and short-term recognition memory in the NOR test) [26,27,28].

An interesting observation reported in the present study and previously [24] concerns a change in the sensitivity of animals to the action of muscarinic ligands after chronic administration of MK-801. The impairments in cognition induced by blockade of NMDA receptors for seven days were reversed by chronic administration of M_1_, M_4_, or M_5_ activators at lower doses than those needed to prevent memory dysfunction induced with acute administration of MK-801. Similar observations were reported earlier for mGlu_2_ activator, LY487379 [24]. The activity of F15599 was similar in both experimental schedules. This finding might be of clinical importance, indicating a low risk of tolerance development and the possibility of decreasing drug dosing with the duration of the treatment.

The next part of investigations aimed to confirm or exclude putative drug–drug interactions that could occur after the combined administration of the compounds.

In contrast to our earlier studies, the analysis was performed in the frontal cortex, the structure postulated to be involved in higher processing in the brain [29,30], with high expression of muscarinic receptors [21,31,32], and the site of the preferential action of the 5-HT_1A_-biased agonist F15599 [19].

Pharmacokinetic studies of F15599 revealed its rapid brain penetration (15 min) with a relatively long half-life in the FC (120 min) and high FC penetrability, as suggested in the literature [19,20]. Both VU0357017 and VU0238429 displayed high FC penetration, reaching 67% and 53%, respectively, and the FC/plasma ratio for VU0152100 was 7%. Compared to earlier studies performed both in our laboratory [21,22,24] and by others [33,34,35] in which the whole-brain penetrability of muscarinic ligands was investigated, the brain/plasma ratios for both VU0357017, an M_1_ allosteric agonist, and VU0152100, an M_4_ PAM, were similar to the FC/plasma ratio [21]. VU0238429, the M_5_ PAM, appears to preferentially accumulate in the FC. Earlier studies indicated relatively weak brain penetration of the compound, which was not higher than 8% [24,34]. The more than 6 times higher penetration to the FC would explain the ability of this compound to reverse MK-801-induced memory impairments, and confirms its putative therapeutic potency.

The acute administration of the combinations at subeffective doses resulted in decreased FC penetration of VU0357017, VU0238429, and F15599 compared to the FC/plasma ratio observed after the acute administration of the top doses of each compound alone. This result potentially indicates possible drug-drug interactions and may explain the lack of interaction in the behavioral tests described above.

Prolonged MK-801 administration decreased brain penetration by up to 50% for all the compounds at the top doses (except VU0152100) compared to single administration. This result contradicts behavioral studies in which lower doses of the compounds were active. Further studies are needed to understand this phenomenon.

The repeated administration of MK-801 and the combinations at subeffective doses did not affect FC penetration properties in comparison to acute administration.

The present results fit the trends indicating the potential of muscarinic receptors as targets for antipsychotic drugs, which were first presented clinically by Shekhar et al. [36] for xanomeline and later developed by Karuna Therapeutics (https://clinicaltrials.gov/ct2/show/NCT03697252, access date: 18 June 2021). Karuna Therapeutics’s KarXT circumvents the adverse effects of xanomeline, as the drug is combined with an FDA-approved muscarinic antagonist (trospium) to reduce off-target activity related to activation of peripheral M_2_ and M_3_ receptors [37,38]. Subsequent studies indicated that the selective activation of M_1_, M_4_, or M_5_ receptors (presumed to be preferentially expressed in the brain [32]) was a better and safer alternative to xanomeline. To date, these and our recent studies are the most relevant confirming the activity of the ligands in animal models of schizophrenia-related cognitive symptoms.

As the brains of patients with schizophrenia display dysfunctions in receptor expression or excitability (for a review, see: [39]), a certain benefit may be the simultaneous stimulation of two different receptors, as proposed in many of our previous papers [39]. As the 5-HT_1A_ receptor is postulated to play an essential role in cognition, here, we show that simultaneous, chronic activation of muscarinic, and 5-HT_1A_ receptors may exert a therapeutic effect on some forms of learning at relatively low doses and offer a possibility to reduce the adverse effects observed for xanomeline or (R,S)-8-OH-DPAT alone, thus eliminating the need to use add-on drugs, e.g., trospium.

**Figure 14:**
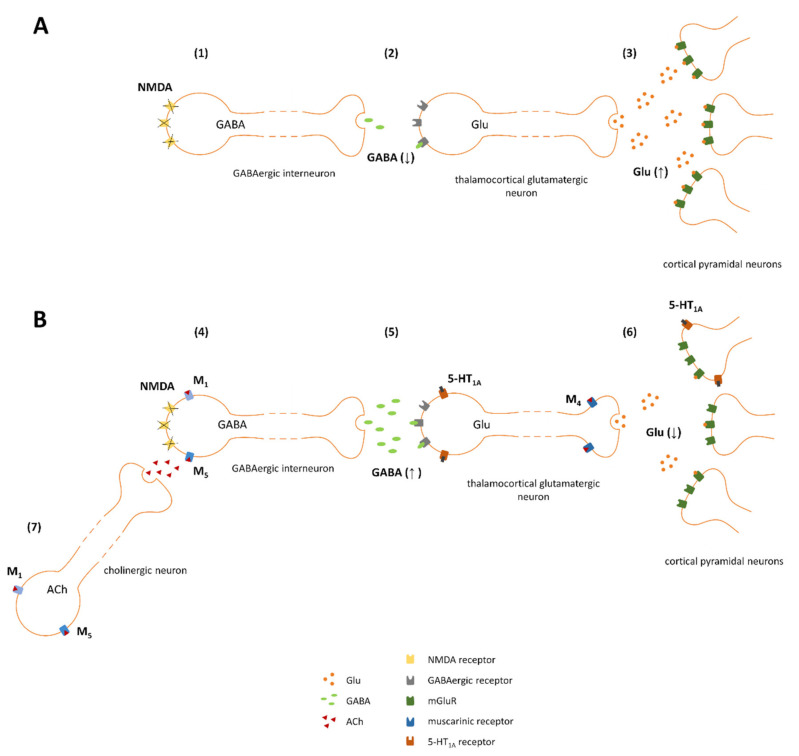
Proposed mechanism of action of investigated combinations (basic scheme adapted from Conn et al. [43]). (**A**) Impaired effect of glutamate on dysfunctional NMDA receptors located on GABAergic neurons (1), results in decreased GABA release (2), disinhibition of subsequent thalamocortical glutamatergic innervation and overactivation of pyramidal glutamatergic neurons in the cortex (3). (**B**) M_1_ and M_5_ receptors may be expressed on GABAergic neurons (4), and thus, their activation by VU0357017 or VU0238429 counteracts NMDA-related impairments (both M_1_ and M_5_ receptors are linked to Gq protein and thus are excitatory in nature); additionally, the ligands may activate M_1_ or M_5_ receptors expressed on cholinergic neurons innervating GABAergic interneurons (7). 5-HT_1A_ receptors are expressed postsynaptically on glutamatergic neurons in the cortex (5). The receptors are linked to Gi proteins; thus, their activation by F15599 inhibits neuronal activity and counteracts the hyperexcitability observed in individuals with schizophrenia. M_4_ receptors are expressed presynaptically on glutamatergic terminals; their stimulation by VU0152100 activates Gi protein, which are inhibitory in nature and leads to the inhibition of glutamate release (6).

To explain the mechanism of action of investigated ligands we followed the hypothesis of NMDA receptor insufficiency as the main disturbance responsible for schizophrenia. The glutamatergic hypothesis of schizophrenia was formulated based on the work of Maria and Arvid Carlsson, who were first to suggest that aside from dopaminergic hyperactivity, glutamatergic dysregulation could be one of the main causes of the disease [40]. The hypothesis assumes that glutamatergic hyperactivity in the brain is associated with the deficiency of NMDA receptors, located on the inhibitory GABAergic interneurons [41,42,43,44,45]. According to this hypothesis disinhibited thalamocortical glutamatergic neurotransmission was the core cause of enhanced release of the other neurotransmitters, including dopamine (increased dopamine release was initially proposed by Carlsson as the main cause of schizophrenia and contributed to the formulation of dopaminergic theory of schizophrenia which was the first hypothesis of the disease introduced in 1977 [46]). Therefore, the inhibition of increased glutamate release in the cortex can be proposed as the primary mechanism of the action of the ligands administered either alone or in combinations (schematically presented on Figure 14). Activation of 5-HT_1A_ receptors expressed postsynaptically on cortical glutamatergic neurons inhibits the activity of those neurons and thus counteracts the glutamatergic hyperexcitability. Additionally, stimulation of M_4_ receptors located presynaptically on glutamatergic terminals leads to the inhibition of excessive glutamate release. Activation of M_1_ or M_5_ receptors expressed on GABAergic interneurons leads to an increase of GABA release and thus may compensate for NMDA receptor-dysfunction. Overall, as the final result of the treatment dopaminergic-glutamatergic balance in the brain should be restored.

## 4. Materials and Methods

### 4.1. Animals and Housing

Male Albino Swiss mice (Charles River, Sulzfeld, Germany) weighing 20–25 g at the beginning of the experiments were used in the studies. The animals were housed in a room with a 12:12 h light-dark cycle at a temperature of 22 ± 1 °C with food and water available ad libitum. The experimental groups consisted of 8–10 animals. All drugs were administered intraperitoneally (i.p.) at a volume of 10 mL/kg. The behavioral experiments were performed by an observer who was blinded to the treatment. The procedures were conducted in accordance with the European Communities Council Directive of 22 September 2010 (2010/63/EU) and Polish legislation acts concerning animal experimentation and were approved by the II Local Ethics Committee by the Maj Institute of Pharmacology, Polish Academy of Sciences in Krakow (15/2020, 240/2020).

### 4.2. Drugs

MK-801 (Hello Bio, Bristol, UK), F15599 (NLX-101) (MedChemExpress) and VU0357017 hydrochloride (Biorbyt) were dissolved in 0.9% NaCl. VU0152100 (Biorbyt) and VU0238429 (Tocris) were dissolved in 10% Tween 80 in 0.9% NaCl. When the administration of the tested compounds was omitted (control and MK-801 groups), the animals received appropriate vehicles. The doses used in behavioral experiments were based on our previous studies [22,23] and available data [25,47,48].

### 4.3. Treatment Regimen

In acute experiments, the tested compounds (alone or in combination) were administered 30 min before MK-801 (0.3 mg/kg), which was administered 30 min before the test or training trial (T_1_) in the NOR test. In chronic experiments, the tested compounds (alone or in combination) and MK-801 were administered for seven days. Behavioral tests were performed on the eighth day. The treatment schedules are schematically presented in Figure 1.

### 4.4. Novel Object Recognition Test

The NOR test was performed as described previously [21,24]. Briefly, the habituation, training and test trial were carried out in a dark room in a black, plastic rectangular arena illuminated with a 355 lux bulb. During the habituation trial, the animals were allowed to explore the arena in the absence of objects (10 min/day for 2 days). The next day, the training (T_1_) and test (T_2_) trials were performed. Throughout T_1_, mice were allowed to freely explore two identical objects (5 min), one of which was subsequently replaced by a novel object in T_2_ (1 h later, 5 min of exploration). Time spent exploring (i.e., sniffing or touching) the familiar (T_familiar_) or novel object (T_novel_) was measured by a trained observer, and then the recognition index was calculated for each mouse [(T_novel_ − T_familiar_)/(T_familiar_ + T_novel_)] × 100.

### 4.5. Y-Maze

The Y-maze was performed using the methods described by Miedel et al. and Kraeuter et al. [26,49] with minor modifications. A black, wooden Y-shaped apparatus with equal length arms (labeled A, B, or C) spaced 120 apart was used in the behavioral experiment. Visual clues were placed inside the maze and mounted to the back wall of each arm. The experiment was performed in a well-lit room. The mice were placed in the same starting arm and were allowed to freely explore the maze for 8 min. After each trial, the maze was wiped clean. The sequence and the number of arm entries were recorded to calculate the percentage of spontaneous alternations using the following formula: [(Number of alternations)/(Total arm entries − 2)] × 100. An arm entry was scored if the animal entered the arm with all paws. Consecutive entries into three distinct arms were scored as an alternation (e.g., ACB, but not ACA) in which sequences could overlap (e.g., ACBACABAC represents five alternations).

### 4.6. Pharmacokinetic Studies

The experiment was performed using the methods described by Bridges et al. [34] and in our previous studies [21,22,24].

The pharmacokinetic analysis was performed in the blood and prefrontal cortex. Blood was collected from the inferior vena cava, transferred to an EDTA-coated tube (Sarstedt) and centrifuged at 3500× *g* rpm for 10 min at RT. Plasma and FC samples were stored at −80 °C until analysis.

#### 4.6.1. Pharmacodynamic Studies of F15599 after Acute Administration

The drug was administered at a dose of 0.1 mg/kg; brain and tissue samples were collected 15, 30, 60, and 120 min after administration.

#### 4.6.2. Pharmacokinetic Studies after Acute Administration of the Combined Treatment

Each compound was administered alone at the top dose (F15599—0.1 mg/kg, VU0357017—5 mg/kg, VU0152100—1 mg/kg, and VU0238429—5 mg/kg) and in combinations of muscarinic ligands at subeffective doses with F15599 at subeffective dose (F15599 + VU0357017 0.05 and 1 mg/kg, F15599 + VU0152100 0.05 and 0.25 mg/kg, F15599 + VU0238429 0.05 and 1 mg/kg). The FC and blood were collected 30 min after administration.

#### 4.6.3. Pharmacokinetic Studies after Prolonged Administration of Combined Treatment

The investigated compounds were administered alone at the top dose (F15599—0.1 mg/kg, VU0357017—5 mg/kg, VU0152100—1 mg/kg, and VU0238429—5 mg/kg) and in combinations of muscarinic ligands at subeffective doses with F15599 at subeffective dose (F15599 + VU0357017 0.05 and 0.15 mg/kg, F15599 + VU0152100 0.05 and 0.05 mg/kg, F15599 + VU0238429 0.05 and 1 mg/kg). The FC and blood were collected on the eighth day, 30 min after administration. All mice were parallelly treated with MK-801 (0.3 mg/kg) for seven consecutive days.

On the day of analysis, frozen whole mouse brain tissues were weighed and homogenized in 1:2 (*w*/*v*) volumes of ice-cold acetonitrile containing 0.1% FA and the internal standard mix. Samples were homogenized using a Metal Bead Lysing Matrix, vortexed for 1 min, shaken for 20 min at 4 °C on Thermomixer C, and centrifuged at 14,000× *g* rpm for 20 min at 4 °C. Finally, 50 µL of supernatant were diluted with 0.1% FA in water (1:1, *v*/*v*) and analyzed using HPLC/MS/MS.

The sample extraction of plasma (20 μL) was performed after spiking with the internal standard mix using the protein precipitation method and three volumes of ice-cold acetonitrile containing 0.1% formic acid. The extract was vortexed, incubated on ice for 20 min and centrifuged at 14,000× *g* rpm for 20 min at 4 °C. The supernatant was diluted with 0.1% FA in water (1:1, *v*/*v*) and analyzed by means of HPLC/MS/MS using a QTRAP 4500 (AB Sciex) mass spectrometer in positive ion mode in Multiple Reaction Monitoring (MRM screening) coupled with UHPLC (NEXERA XR, Shimadzu). Chromatographic separation was achieved on a Synergi 4 µm Fusion-RP 80 A 50 × 2 mm (Phenomenex) column at a flow rate of 0.2 mL/min. The gradient program was as follows: 20% B (0.3 min), 20–100% B (1.7 min), 100% B (3 min), 100–20% B (0.1 min), 20% B (2 min), solvent A (95:5:0.1% formic acid in water:methanol) and solvent B (methanol containing 0.1% formic acid). The column temperature was set to 40 °C. Analyst software was used to control the instrument and collect data. The ion transfer tube temperature was 300 °C. The spray voltage, collision energy, declastering potential, and gas parameters were optimized to achieve the maximal response using the test compounds. Selected reaction monitoring was carried out using the transition parameters. Calibration curves were constructed, and a linear response was obtained in the range of 0.25–500 ng/mL (VU357017, VU0152100, and F15599) and 1.25–2500 ng/mL (VU0238429) by spiking known amounts of each compound in blank brain homogenates and plasma. For VU0357017 and F15599 IS VU0152100 was used as internal standard. Sample concentration of internal standard was 100 ng/mL for IS VU0238429 and 50 ng/mL for IS VU0152100.

Brain penetration was calculated as FC/plasma ratio × 100%.

### 4.7. Statistics

The statistical analysis was performed using GraphPad Prism v.9.1.0 or TIBCO Statistica 13.3 software. The results of the dose-dependent studies were analyzed with one-way ANOVA followed by Dunnett’s post hoc comparison. The results of interaction studies were analyzed with two-way ANOVA followed by Tukey’s post hoc for unequal N comparison.

### 4.8. Software

Marvin JS (ChemAxon, https://chemaxon.com/products/marvin-js, access date: 1 August 2021) was used to draw chemical structures in Figure 1.

## 5. Conclusions

The present studies show the potential benefit of using muscarinic and serotonergic ligands in the treatment of cognitive symptoms of schizophrenia. The activation of muscarinic M_1_, M_4_, and M_5_ receptors prevented MK-801-induced memory disruption in NOR and Y-maze tests both after acute and chronic administration of their activators, VU0357017, VU0152100, and VU0238429, respectively. The efficacy of the compounds was observed at much lower doses after prolonged administration when compared to acute treatment and this was accompanied with better FC penetration. Such discrepancy was not observed in the case of 5-HT_1A_ activator, F15599, preferentially binding to the receptors expressed in the frontal cortex.

Simultaneous acute administration of the non-effective doses of the muscarinic activators with F15599 was not effective in neither NOR or Y-maze test. In contrast, prolonged simultaneous administration of the subeffective doses of the compounds along with MK-801 prevented MK-801-induced memory disruption at the same way as the top doses given alone. Pharmacokinetic analysis revealed decreased FC penetration of the compounds after simultaneous, acute administration, and may suggest on some drug–drug interaction that might influence drugs’ efficacies. Such an effect was not observed after prolonged administration of the combinations. No synergistic activity of the combinations was observed in Y-maze, neither after acute nor prolonged administration.

Taken together the proposed treatment, based on the chronic administration of the muscarinic ligands with 5-HT1A agonist may be proposed for the treatment of cognitive decline observed in schizophrenic patients. Importantly, much lower doses of the compounds are needed for chronic treatment to induce therapeutic effect, which limits the possibility to induce adverse effects or development of tolerance. Moreover, combined treatment can be more effective when the expression or the function of particular receptor subtypes is disturbed due to pathological state, as the compounds can compensate each other’s action.

## Figures and Tables

**Table 1:** Pharmacokinetic analysis of the FC penetration of the drugs administered at the highest doses alone and the combined administration of the compounds at the subeffective doses. The compounds were administered both acutely and for seven days along with MK-801.

Acute Administration	Plasma (ng/mL)	PFC (ng/mL)	PFC Penetration (%)	Chronic Administration	Plasma (ng/mL)	PFC (ng/mL)	PFC Penetration (%)
**Top doses**				**Top doses**			
VU0357017 (5 mg/kg)	107.28 ± 7.65	71.65 ± 4.69	67%	VU0357017 (5 mg/kg)	138.15 ± 14.91	50.33 ± 4.62	36%
VU0152100 (1 mg/kg)	78.44 ± 11.86	5.71 ± 1.51	7%	VU0152100 (1 mg/kg)	60.91 ± 2.66	7.85 ± 0.53	13%
VU0238429 (5 mg/kg)	486 ±178	256 ± 32.06	53%	VU0238429 (5 mg/kg)	844.14 ± 235	179.20 ± 24.98	21%
F15599 (0.1 mg/kg)	2.95 ± 0.4	18.29 ± 0.84	620%	F15599 (0.1 mg/kg)	6.98 ± 0.7	21.57 ± 1.26	309%
**Subeffective doses**				**Subeffective doses**			
F15599 (0.05 mg/kg) VU0357017 (1 mg/kg)	1.77 ± 0.24	11.26 ± 1.25	636%	F15599 (0.05 mg/kg) VU0357017 (0.15 mg/kg)	1.9 ± 0.23	18.01 ± 2.23	948%
	7.19 ± 0.24	2.25 ± 0.56	31%		5.13 ± 0.38	2.34 ± 0.255	46%
F15599 (0.05 mg/kg) VU0152100 (0.25 mg/kg)	5.32 ± 0.39	18.15 ± 0.74	341%	F15599 (0.05 mg/kg) VU0152100 (0.05 mg/kg)	6.86 ± 0.38	32.12 ± 1.27	468%
	7.62 ± 0.63	1.4 ± 0.11	18%		3.77 ± 0.26	0.54 ± 0.07	14%
F15599 (0.05 mg/kg) VU0238429 (1 mg/kg)	5.1 ± 0.37	16.58 ± 0.53	325%	F15599 (0.05 mg/kg) VU0238429 (1 mg/kg)	6.5 ± 0.44	32.35 ± 1.14	498%
	119.91 ± 13.9	43.12 ± 1.81	36%		35 ± 8.89	5.13 ± 0.83	15%

## References

[1] Sheffield J.M., Karcher N.R., Barch D.M. (2018). Cognitive Deficits in Psychotic Disorders: A Lifespan Perspective. Neuropsychol. Rev..

[2] Owen M.J., Sawa A., Mortensen P.B. (2016). Schizophrenia. Lancet.

[3] Zanelli J., Mollon J., Sandin S., Morgan C., Dazzan P., Pilecka I., Marques T.R., David A.S., Morgan K., Fearon P. (2019). Cognitive change in schizophrenia and other psychoses in the decade following the first episode. Am. J. Psychiatry.

[4] Millan M.J., Agid Y., Brüne M., Bullmore E.T., Carter C.S., Clayton N.S., Connor R., Davis S., Deakin B., Derubeis R.J. (2012). Cognitive dysfunction in psychiatric disorders: Characteristics, causes and the quest for improved therapy. Nat. Rev. Drug Discov..

[5] Mauri M.C., Paletta S., Maffini M., Colasanti A., Dragogna F., Di Pace C., Altamura A.C. (2014). Clinical pharmacology of atypical antipsychotics: An update. EXCLI J..

[6] Horiguchi M., Miyauchi M., Neugebauer N.M., Oyamada Y., Meltzer H.Y. (2016). Prolonged reversal of the phencyclidine-induced impairment in novel object recognition by a serotonin (5-HT)1A-dependent mechanism. Behav. Brain Res..

[7] Wierońska J.M., Sławińska A., Łasoń-Tyburkiewicz M., Gruca P., Papp M., Zorn S.H., Doller D., Kłeczek N., Noworyta-Sokołowska K., Gołembiowska K. (2015). The antipsychotic-like effects in rodents of the positive allosteric modulator Lu AF21934 involve 5-HT1A receptor signaling: Mechanistic studies. Psychopharmacology.

[8] Nagai T., Murai R., Matsui K., Kamei H., Noda Y., Furukawa H., Nabeshima T. (2009). Aripiprazole ameliorates phencyclidine-induced impairment of recognition memory through dopamine D1 and serotonin 5-HT1A receptors. Psychopharmacology.

[9] Mutlu O., Ulak G., Celikyurt I.K., Akar F.Y., Erden F., Tanyeri P. (2011). Effects of olanzapine, sertindole and clozapine on MK-801 induced visual memory deficits in mice. Pharmacol. Biochem. Behav..

[10] Ohno Y. (2011). Therapeutic Role of 5-HT1A Receptors in The Treatment of Schizophrenia and Parkinson’s Disease. CNS Neurosci. Ther..

[11] Meltzer H.Y., Horiguchi M., Massey B.W. (2011). The role of serotonin in the NMDA receptor antagonist models of psychosis and cognitive impairment. Psychopharmacology.

[12] Meltzer H.Y., Rajagopal L., Huang M., Oyamada Y., Kwon S., Horiguchi M. (2013). Translating the N-methyl-d-aspartate receptor antagonist model of schizophrenia to treatments for cognitive impairment in schizophrenia. Int. J. Neuropsychopharmacol..

[13] Wierońska J.M., Acher F.C., Sławińska A., Gruca P., Łasoń-Tyburkiewicz M., Papp M., Pilc A. (2013). The antipsychotic-like effects of the mGlu group III orthosteric agonist, LSP1-2111, involves 5-HT1A signalling. Psychopharmacology.

[14] Woźniak M., Gołembiowska K., Noworyta-Sokołowska K., Acher F., Cieślik P., Kusek M., Tokarski K., Pilc A., Wierońska J.M. (2017). Neurochemical and behavioral studies on the 5-HT1A-dependent antipsychotic action of the mGlu4 receptor agonist LSP4-2022. Neuropharmacology.

[15] Wierońska J.M., Sławińska A., Stachowicz K., Łasoń-Tyburkiewicz M., Gruca P., Papp M., Pilc A. (2013). The reversal of cognitive, but not negative or positive symptoms of schizophrenia, by the mGlu2/3 receptor agonist, LY379268, is 5-HT1A dependent. Behav. Brain Res..

[16] Newman-Tancredi A. (2011). Biased agonism at serotonin 5-HT _1A_ receptors: Preferential postsynaptic activity for improved therapy of CNS disorders. Neuropsychiatry.

[17] Celada P., Bortolozzi A., Artigas F. (2013). Serotonin 5-HT1Areceptors as targets for agents to treat psychiatric disorders: Rationale and current status of research. CNS Drugs.

[18] Ögren S.O., Eriksson T.M., Elvander-Tottie E., D’Addario C., Ekström J.C., Svenningsson P., Meister B., Kehr J., Stiedl O. (2008). The role of 5-HT1A receptors in learning and memory. Behav. Brain Res..

[19] Newman-Tancredi A., Martel J.C., Assié M.B., Buritova J., Lauressergues E., Cosi C., Heusler P., Bruins Slot L., Colpaert F.C., Vacher B. (2009). Signal transduction and functional selectivity of F15599, a preferential post-synaptic 5-HT 1A receptor agonist. Br. J. Pharmacol..

[20] Becker G., Bolbos R., Costes N., Redouté J., Newman-Tancredi A., Zimmer L. (2016). Selective serotonin 5-HT1A receptor biased agonists elicit distinct brain activation patterns: A pharmacoMRI study. Sci. Rep..

[21] Cieślik P., Domin H., Chocyk A., Gruca P., Litwa E., Płoska A., Radulska A., Pelikant-Małecka I., Brański P., Kalinowski L. (2020). Simultaneous activation of mGlu2 and muscarinic receptors reverses MK-801-induced cognitive decline in rodents. Neuropharmacology.

[22] Cieślik P., Woźniak M., Tokarski K., Kusek M., Pilc A., Płoska A., Radulska A., Pelikant-Małecka I., Żołnowska B., Sławiński J. (2019). Simultaneous activation of muscarinic and GABA B receptors as a bidirectional target for novel antipsychotics. Behav. Brain Res..

[23] Cieślik P., Woźniak M., Rook J.M., Tantawy M.N., Conn P.J., Acher F., Tokarski K., Kusek M., Pilc A., Wierońska J.M. (2018). Mutual activation of glutamatergic mGlu4 and muscarinic M4 receptors reverses schizophrenia-related changes in rodents. Psychopharmacology.

[24] Cieślik P., Radulska A., Pelikant-Małecka I., Płoska A., Kalinowski L., Wierońska J.M. (2019). Reversal of MK-801-Induced Disruptions in Social Interactions and Working Memory with Simultaneous Administration of LY487379 and VU152100 in Mice. Int. J. Mol. Sci..

[25] Depoortère R., Auclair A.L., Bardin L., Colpaert F.C., Vacher B., Newman-Tancredi A. (2010). F15599, a preferential post-synaptic 5-HT 1A receptor agonist: Activity in models of cognition in comparison with reference 5-HT 1A receptor agonists. Eur. Neuropsychopharmacol..

[26] Kraeuter A.K., Guest P.C., Sarnyai Z. (2019). The Y-Maze for Assessment of Spatial Working and Reference Memory in Mice. Methods in Molecular Biology.

[27] Antunes M., Biala G. (2012). The novel object recognition memory: Neurobiology, test procedure, and its modifications. Cogn. Process..

[28] Bevins R.A., Besheer J. (2006). Object recognition in rats and mice: A one-trial non-matching-to-sample learning task to study “recognition memory”. Nat. Protoc..

[29] Dalley J.W., Cardinal R.N., Robbins T.W. (2004). Prefrontal executive and cognitive functions in rodents: Neural and neurochemical substrates. Neurosci. Biobehav. Rev..

[30] Frith C., Dolan R. (1996). The role of the prefrontal cortex in higher cognitive functions. Cogn. Brain Res..

[31] Levey A.I., Kitt C.A., Simonds W.F., Price D.L., Brann M.R. (1991). Identification and localization of muscarinic acetylcholine receptor proteins in brain with subtype-specific antibodies. J. Neurosci. Off. J. Soc. Neurosci..

[32] Levey A.I. (1993). Immunological localization of m1-m5 muscarinic acetylcholine receptors in peripheral tissues and brain. Life Sci..

[33] Brady A.E., Jones C.K., Bridges T.M., Kennedy J.P., Thompson A.D., Heiman J.U., Breininger M.L., Gentry P.R., Yin H., Jadhav S.B. (2008). Centrally Active Allosteric Potentiators of the M4 Muscarinic Acetylcholine Receptor Reverse Amphetamine-Induced Hyperlocomotor Activity in Rats. J. Pharmacol. Exp. Ther..

[34] Bridges T.M., Marlo J.M., Niswender C.M., Jones C.K., Jadhav S.B., Gentry P.R., Plumley H.C., Weaver C.D., Conn P.J., Lindsley C.W. (2009). Discovery of the First Highly M5-Preferring Muscarinic Acetylcholine Receptor Ligand, an M5 Positive Allosteric Modulator Derived from a Series of 5-Trifluoromethoxy N-Benzyl Isatins. J. Med. Chem..

[35] Lebois E.P., Bridges T.M., Lewis L.M., Dawson E.S., Kane A.S., Xiang Z., Jadhav S.B., Yin H., Kennedy J.P., Meiler J. (2010). Discovery and characterization of novel subtype-selective allosteric agonists for the investigation of M1 receptor function in the central nervous system. ACS Chem. Neurosci..

[36] Shekhar A., Potter W.Z., Lightfoot J., Lienemann J., Dubé S., Mallinckrodt C., Bymaster F.P., McKinzie D.L., Felder C.C. (2008). Selective muscarinic receptor agonist xanomeline as a novel treatment approach for schizophrenia. Am. J. Psychiatry.

[37] Yohn S.E., Conn P.J. (2018). Positive allosteric modulation of M1 and M4 muscarinic receptors as potential therapeutic treatments for schizophrenia. Neuropharmacology.

[38] Langmead C.J., Watson J., Reavill C. (2008). Muscarinic acetylcholine receptors as CNS drug targets. Pharmacol. Ther..

[39] Cieślik P., Wierońska J.M. (2020). Regulation of glutamatergic activity via bidirectional activation of two select receptors as a novel approach in antipsychotic drug discovery. Int. J. Mol. Sci..

[40] Carlsson M., Carlsson A. (1990). Schizophrenia: A Subcortical Neurotransmitter Imbalance Syndrome?. Schizophr. Bull..

[41] Moghaddam B., Adams B., Verma A., Daly D. (1997). Activation of glutamatergic neurotransmission by ketamine: A novel step in the pathway from NMDA receptor blockade to dopaminergic and cognitive disruptions associated with the prefrontal cortex. J. Neurosci..

[42] Olney J.W., Farber N.B. (1995). Glutamate receptor dysfunction and schizophrenia. Arch. Gen. Psychiatry.

[43] Conn P.J., Lindsley C.W., Jones C.K. (2009). Activation of metabotropic glutamate receptors as a novel approach for the treatment of schizophrenia. Trends Pharmacol. Sci..

[44] Moghaddam B. (2004). Targeting metabotropic glutamate receptors for treatment of the cognitive symptoms of schizophrenia. Psychopharmacology.

[45] Javitt D.C., Zukin S.R. (1991). Recent Advances in the Phencyclidine Model of Schizophrenia. Am. J. Psychiatry.

[46] Carlsson A. (1977). Does dopamine play a role in schizophrenia?. Psychol. Med..

[47] Jastrzębska-Więsek M., Partyka A., Rychtyk J., Śniecikowska J., Kołaczkowski M., Wesołowska A., Varney M.A., Newman-Tancredi A. (2018). Activity of Serotonin 5-HT1A Receptor Biased Agonists in Rat: Anxiolytic and Antidepressant-like properties. ACS Chem. Neurosci..

[48] Assié M.B., Bardin L., Auclair A.L., Carilla-Durand E., Depoortère R., Koek W., Kleven M.S., Colpaert F., Vacher B., Newman-Tancredi A. (2010). F15599, a highly selective post-synaptic 5-HT1A receptor agonist: In-vivo profile in behavioural models of antidepressant and serotonergic activity. Int. J. Neuropsychopharmacol..

[49] Miedel C.J., Patton J.M., Miedel A.N., Miedel E.S., Levenson J.M. (2017). Assessment of Spontaneous Alternation, Novel Object Recognition and Limb Clasping in Transgenic Mouse Models of Amyloid-β and Tau Neuropathology. J. Vis. Exp..

